# Thermal and Mechanical Properties of the Biocomposites of *Miscanthus* Biocarbon and Poly(3-*H*ydroxybutyrate-*co*-3-*H*ydroxyvalerate) (PHBV)

**DOI:** 10.3390/polym12061300

**Published:** 2020-06-06

**Authors:** Zonglin Li, Christoff Reimer, Tao Wang, Amar K. Mohanty, Manjusri Misra

**Affiliations:** 1 Bioproducts Discovery and Development Centre, Department of Plant Agriculture, Crop Science Building, University of Guelph, Guelph, ON N1G 2W1, Canada; leezonglin@126.com (Z.L.); reimerc@uoguelph.ca (C.R.); taowang@uoguelph.ca (T.W.); 2 School of Engineering, Thornbrough Building, University of Guelph, Guelph, ON N1G 2W1, Canada

**Keywords:** biocarbon, *Miscanthus*, PHBV, biocomposite, mechanical properties, impact strength, crystallinity, thermal stability, CLTE

## Abstract

*Miscanthus* biocarbon (MB), a renewable resource-based, carbon-rich material, was melt-processed with poly (3-hydroxybutyrate-*co*-3-hydroxyvalerate) (PHBV) to produce sustainable biocomposites. The addition of the biocarbon improved the Young’s modulus of PHBV from 3.6 to 5.2 GPa at 30 wt % filler loading. An increase in flexural modulus, up to 48%, was also observed. On the other hand, the strength, elongation-at-break and impact strength decreased. Morphological study of the impact-fractured surfaces showed weak interaction at the interface and the existence of voids and agglomerates, especially with high filler contents. The thermal stability of the PHBV/MB composites was slightly reduced compared with the neat PHBV. The biocarbon particles were not found to have a nucleating effect on the polymer. The degradation of PHBV and the formation of unstable imperfect crystals were revealed by differential scanning calorimetry (DSC) analysis. Higher filler contents resulted in reduced crystallinity, indicating more pronounced effect on polymer chain mobility restriction. With the addition of 30 wt % biocarbon, the heat deflection temperature (HDT) became 13 degrees higher and the coefficient of linear thermal expansion (CLTE) decreased from 100.6 to 75.6 μm/(m·°C), desired improvement for practical applications.

## 1. Introduction

With increasing concerns over limited fossil-fuel reserves and the burden of human activities on the environment around the world, substantial opportunities for biobased and biodegradable plastics and composites have emerged [1]. Among the most popular bioplastics are the polyhydroxyalkanoates (PHAs), a family of biopolyesters that can be synthesized by microorganisms from renewable resources [2]. Poly (3-hydroxybutyrate) (PHB) is one of the most popular PHAs and is being tested for packaging and biomedical applications [3,4]. PHB is extremely brittle and can suffer thermal degradation during processing [5]. Poly (3-hydroxybutyrate-*co*-3-hydroxyvalerate) (PHBV) is a copolymer composed primarily of hydroxybutyrate units, with randomly distributed hydroxyvalerate (HV) units integrated into the backbone. Compared with PHB, PHBV has decreased brittleness and a wider processing window [6]. PHBV is biocompatible and has good gas barrier properties among biobased polymers [7]. It has also shown great potential in its recyclability, with the capacity for being processed multiple times with minimal change in its material properties [8].

However, the use of PHBV in practical applications necessitates further improvement to its mechanical and thermal properties, such as strength, toughness, thermal stability and dimensional stability at high temperatures [9]. Various strategies have been investigated, including blending with other polymers and preparing composites with a wide range of fillers, ranging from natural fibers, to mineral particles, to nanomaterials [7,10]. In the present work, we explore the use of biocarbon, a carbon-rich material obtained by the incomplete combustion of biomass, as a filler in PHBV. Recent studies have demonstrated that biocarbon can be a renewable alternative to carbon black, which is widely used in the industry as a colorant and filler in automotive parts and rubber products [11,12]. Biocarbon is a promising option for entrapping carbon, and provides numerous opportunities for use in a variety of sectors, notably in soil amendment, catalyst, energy storage devices, and polymer composites [13,14,15,16]. It is a versatile material because it can be chemically modified. One example is the grafting of functional groups onto its surface for enhanced compatibility with polymers [17].

Another major drawback of PHBV is its relatively high cost of production [18]. The incorporation of biocarbon can reduce the overall polymer content in the composite and may also provide a reinforcing effect [19]. Creating biocarbon-filled biopolymer composites provides an opportunity to reduce our reliance on petroleum resources.

The addition of biocarbon can have a significant impact on the performances of the composites. Ho et al. studied bamboo-biocarbon-filled poly(lactic acid) (PLA) composites, and found that the tensile and flexural strength of the composites increased gradually up to a filler content of 7.5 wt %, but decreased at 10 wt % [20]. The onset degradation temperature of the composites was lower than that of the neat polymer, indicating slight decrease of thermal stability. In a different work, the addition of 20 wt % biocarbon obtained from peanut hull reduced both the tensile strength and impact strength of poly (trimethylene terephthalate) (PTT), and the effect was attributed to the biocarbon’s wide size distribution and the poor filler-matrix interaction [21]. A recent study by Arrigo et al. involved the preparation of poly (lactic acid) (PLA)-based composites filled with biocarbon from spent ground coffee, by both melt-blending and solvent casting [22]. The processing techniques were found to have significant influence on the filler dispersion, the crystallization and rheological behavior, and in turn the thermal and mechanical properties.

The biocarbon chosen for the present work is derived from *Miscanthus*, which is a high-yield and hardy perennial grass requiring low maintenance [23]. Furthermore, *Miscanthus* biocarbon has relatively high surface area, among biocarbon materials produced from different biomass feedstock [24]. *Miscanthus* biocarbon has been tested as a carbon source for biologically derived graphite and supercapacitor [25,26]. It has also been evaluated as a filler in composites with polymers such as polyamide and polypropylene [27,28,29]. The incorporation of *Miscanthus* biocarbon materials with different particle sizes and shapes, and obtained at different pyrolysis temperatures, was found to affect the mechanical properties, impact strength, heat defection temperature (HDT) and thermal expansion. There have been very few reports on the use of *Miscanthus* biocarbon in biopolymer-based composites. Snowdon et al. compared the effect of adding talc vs. *Miscanthus* biocarbon in PLA, with and without plasticization using poly (ethylene glycol) [30]. They found that the addition of talc decreased the coefficient of linear thermal expansion (CLTE), and the addition of biocarbon increased the value. While talc worked as a nucleating agent for PLA, the biocarbon inhibited crystal growth. In a more recent study by the same authors, unlike talc, biocarbon did not help with reducing the oxygen permeability, and also lowered the glass transition temperature and the onset degradation temperature of the polymer [31]. On the other hand, the biocarbon enhanced the flame resistance by reducing the burning rate of PLA.

This study is aimed at understanding the effect of incorporating biocarbon on the mechanical and thermal properties of PHBV. The composites of PHBV filled with *Miscanthus* biocarbon (PHBV/MB), at 10, 20 and 30 wt % levels, were fabricated, and their tensile, flexural and impact properties were characterized. Morphological comparisons of the fracture surfaces of the neat polymer and the composites helped to elucidate the mechanical behavior observed. The influence of biocarbon addition on the polymer crystallization, thermal stability, and dimensional stability at high temperatures was also investigated.

## 2. Materials and Methods

### 2.1. Materials

The biocarbon was provided by Competitive Green Technologies, Leamington, ON, Canada. In the production of the biocarbon, the *Miscanthus* harvested in Ontario was treated in a continuous pyrolysis equipment in a nitrogen environment at 600 to 650 °C for 10–20 min until the biomass was fully charred. The material obtained was ground with a hammer mill to pass a 1/64 inch (0.4 mm) screen [11]. PHBV (ENMAT Y1000P injection grade), with a density of 1.25 g/cm^3^, was procured from Tianan Biologic Materials, Ningbo, Zhejiang, China.

### 2.2. Composite Preparation

The as-received PHBV pellets were dried in an 80 °C oven for 4 h and the biocarbon was dried at 105 °C for 72 h. The moisture contents of PHBV and biocarbon were measured to be 0.2% and 0.9%, respectively. The PHBV/MB composites were made using a 15 mL micro twin-screw compounder manufactured by Xplore Instruments BV, Sittard, The Netherlands. Extrusion was carried out at 180 °C and 100 rpm screw speed, with retention time of 2 min. Sample bars were produced by using the Xplore mini injection molder with mold temperature set at 30 °C. Composites with filler contents of 10%, 20% and 30% by weight were prepared.

### 2.3. Characterization

A universal testing machine (Model 3382 of Instron, Norwood, MA, USA) was used for tensile and flexural property testing. ASTM D638 was followed for the tensile test, at a speed of 5 mm/min. Flexural modulus and strength were tested based on ASTM D790, with a span distance of 52 mm and a loading rate of 1.4 mm/min. Five replicates were tested for each material formulation. The data was analyzed using the BlueHill software provided by Instron.

A TMI Monitor impact tester (Testing Machines Inc., New Castle, DE, USA) was used to test the notched Izod impact strengths of the PHBV and PHBV/MB composites conforming to ASTM D256. The samples were notched with a notching cutter and tested 40 h after they were prepared. Five replicates were made and the average value and standard deviation were calculated. 

The morphology of the PHBV and PHBV/MB composites was observed using a Phenom ProX scanning electron microscope of Thermo Fisher Scientific Inc., Waltham, MA, USA. The impact-fractured surfaces were gold coated before being imaged at 10 KV accelerating voltage.

The thermal behavior of the materials was studied using Q200 differential scanning calorimeter of TA Instruments, New Castle, DE, USA. The specimens with weight between 5 and 8 mg were loaded in sealed aluminum pans. They were heated from 0 to 190 °C at a rate of 10 °C/min in nitrogen and held for 3 min. The specimens were then cooled to 0 °C at 10 °C/min. The degree of crystallinity, *χ_c_* (%), is calculated with the following equation:

$$\chi_{c}=\frac{\Delta H_{m}}{\Delta H_{o}}\times \frac{1}{w}\times 100\%$$

where ∆*H_m_* is the specific melting enthalpy measured (J/g), *w* is the weight content of PHBV, and ∆*H_o_* is the theoretical 100% crystalline PHB’s melting enthalpy, which has been reported to be 146 J/g [32,33].

Thermogravimetric analysis (TGA) of the PHBV and composites was conducted using TA Instruments Q500 analyzer (TA Instruments). Each specimen (about 10 mg) was heated from room temperature to 600 °C at a rate of 10 °C/min in a nitrogen environment.

The heat deflection temperature (HDT) was measured according to ASTM Standard Method D648, using a dynamic mechanical analyzer, TA Instruments Q800 (TA Instruments), operated in three-point bending mode. Each sample was equilibrated at 30 °C and then heated up at a rate of 2 °C /min, under a load of 0.455 MPa. When the strain reached 0.1889%, the temperature was reported as the HDT. 

A TA Instruments Q400 thermomechanical analyzer (TA Instruments) was used to test the coefficient of linear thermal expansion (CLTE), based on ASTM E831. The samples were tested with an expansion probe (0.1 N) in the injection flow direction. They were heated from 20 to 120 °C at 5 °C /min. The CLTE was calculated using the expansion between 30 and 80 °C.

## 3. Results

### 3.1. Mechanical Properties

**Figure 1:**
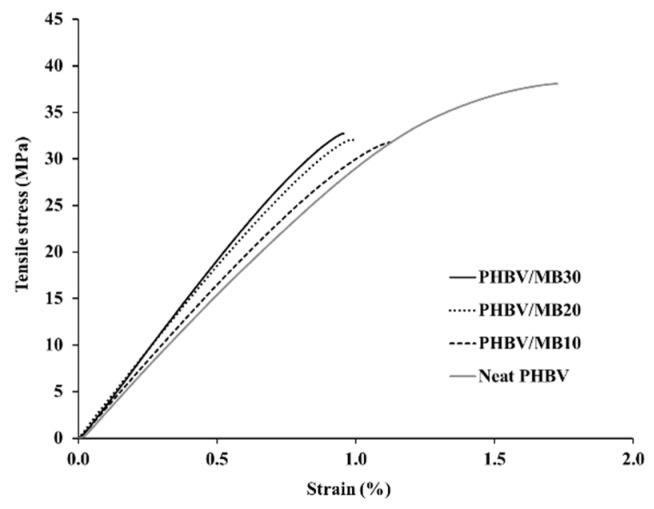
Typical stress-strain curves obtained by tensile test of the PHBV and PHBV/MB composites.

**Figure 2:**
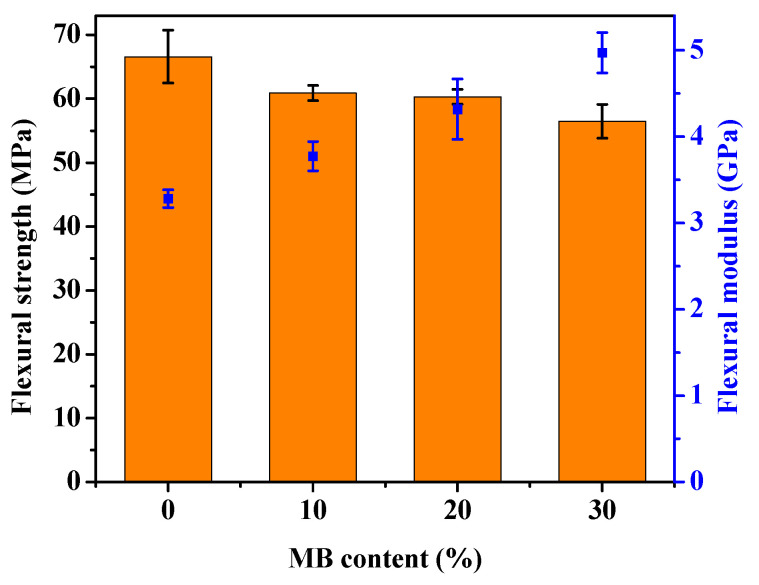
Flexural strength (bars) and modulus (dots) of the PHBV and PHBV/MB composites.

The stress-strain curves obtained from the tensile test of the PHBV and its composites are shown in Figure 1, with the modulus, strength and elongation-at-break results listed in Table 1. The addition of the *Miscanthus* biocarbon increased the tensile modulus of PHBV. The increase grew with higher contents of the biocarbon. At 30 wt % filler loading, the Young’s modulus became 46% higher than that of PHBV. However, the tensile strength decreased with the incorporation of the biocarbon. From Figure 1, it can be seen that the neat PHBV and the composites all showed brittle fracturing, with no yielding behavior. The failure of the composites occurred at even lower strain than the neat PHBV, resulting in a decreased tensile toughness and elongation-at-break with higher contents of biocarbon. Figure 2 shows that there was also a great increase of the flexural modulus with increasing biocarbon loadings, and at the same time a decrease of flexural strength. A similar effect on mechanical properties was reported previously in poly (vinyl alcohol) composites filled with hardwood biocarbon, and polypropylene (PP) composites filled with both *Miscanthus* biocarbon and date palm (*Phoenix dactylifera*) biocarbon [15,29,34]. Biocarbon obtained from lignocellulosic materials is known to have high stiffness and hardness [35]. The addition of high-stiffness particles can improve the modulus of polymers [30,36]. On the other hand, the rigid filler particles cannot be deformed by external stress the same way as the polymer. They have a drastically different elastic response to the polymer matrix around them, and can act as stress concentrators when a load is applied [37]. The addition of the rigid biocarbon particles and their weak interaction with the polymer matrix, as further evidenced by the morphology analysis later, causes stress concentration and ineffective stress transfer, resulting in reduced strength and tensile toughness.

**Figure 3:**
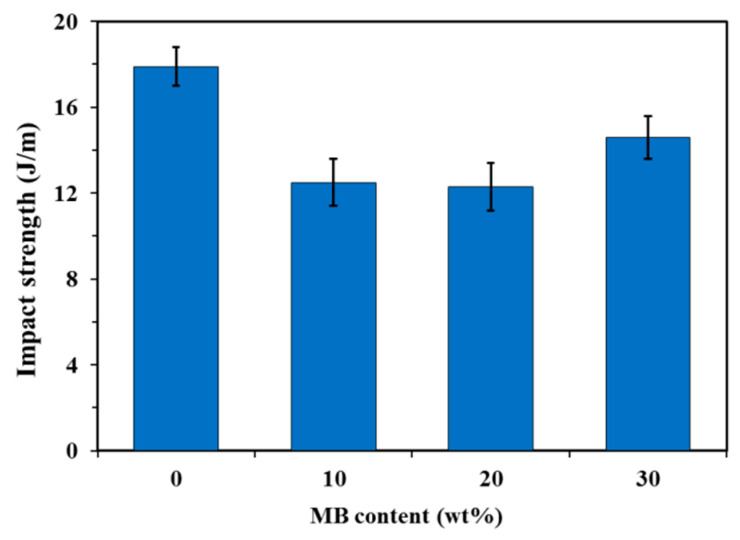
Notched Izod impact strength of the PHBV and PHBV/MB composites.

**Figure 4:**
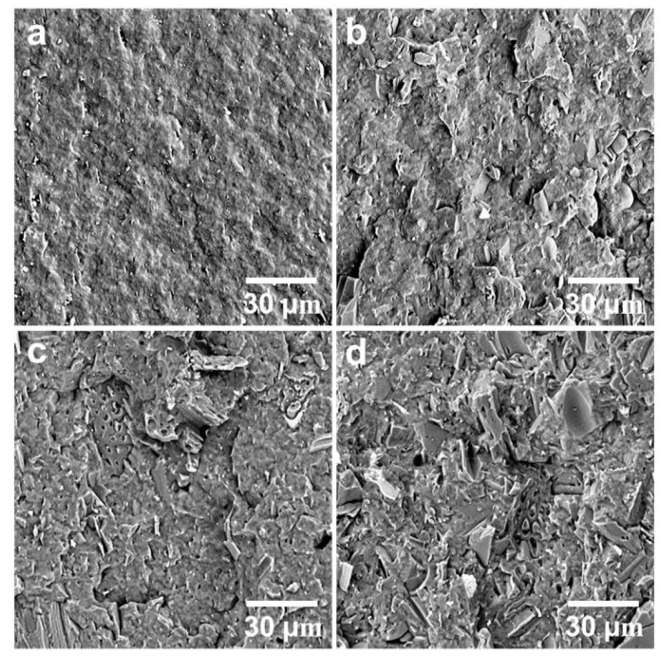
SEM images of the fracture surfaces of (**a**) PHBV, (**b**) PHBV/MB10, (**c**) PHBV/MB20, and (**d**) PHBV/MB30 composites.

The neat PHBV showed higher notched Izod impact strength than the three composites (Figure 3). The addition of the biocarbon particles reduced the material’s capability to absorb impact energy. The restriction of the polymer chain movement by the particles hampers the polymer’s ability to plastically deform to absorb and disperse energy efficiently. Although the impact strength of the MB30 composite was still lower than that of the neat PHBV, it showed a small increase from that of the MB10 and MB20 composites (about 18%). On the fracture surfaces created by impact testing (Figure 4), the MB30 composite showed prevalent agglomeration and pull-out of the particles. The debonding at the filler–matrix interface and the pull-out of the particles may have helped with releasing the stress locally when subjected to impact force [38]. The particle pull-out also creates a roughened surface, which has higher relative surface area than a smooth one. This may also help with impact energy dissipation [39]. All these effects may have moderately mitigated the impact strength reduction of the polymer with the addition of the rigid particles.

### 3.2. Fracture Surfaces

The fracture surfaces of the neat polymer and the composites, created by impact testing, were observed using a scanning electron microscope (Figure 4). The surface of PHBV is smooth, which is typical of a brittle fracture. It indicates weak resistance to crack initiation and propagation. The biocarbon used in the present study was pyrolyzed from *Miscanthus* and milled to pass a 0.4 mm screen. The particles have been characterized in a previous study and found to have irregular shape and large particle size distribution [11]. With increasing amounts of biocarbon added, the surfaces of the composites become rough, indicating that the fillers are not wetted well by the polymer. It also suggests that the addition of the biocarbon can impact the propagation and the advancing of the cracks at the interface between PHBV and the filler. The random distribution of the biocarbon particles in PHBV is observed in the PHBV/MB10 and PHBV/MB20, with the PHBV/MB30 showing increased particle agglomeration. In the PHBV/MB30, the inter-particle distance has become very small. “Clean” particles, with well-defined edges, can be easily distinguished on the surface, indicating particle pull-out.

Two types of voids can be observed. Biocarbon is known to be a porous material [40,41]. Some macro pores are still clearly visible in the large particles on the surfaces of the PHBV/MB20 and PHBV/MB30. The molten polymer did not gain access to these pores during melt-processing. The second type of void is the gap between the particle and polymer matrix. With the biocarbon content going from 10% to 30%, the voids between the particles and the PHBV matrix become more prominent. These defects can contribute to the effect of reduced strength that was observed earlier.

### 3.3. Thermal Behavior

**Figure 5:**
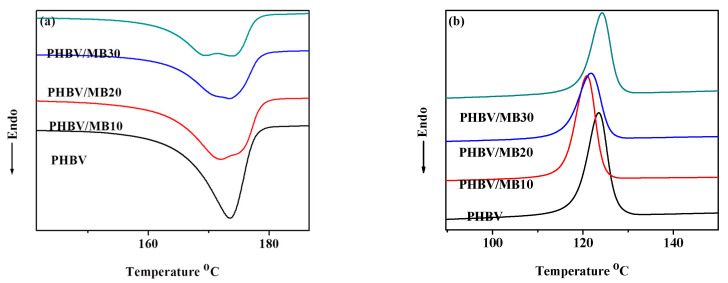
DSC thermograms of the PHBV and PHBV/MB composites during: (**a**) first heating run; (**b**) first cooling run.

The differential scanning calorimetry (DSC) curves of the PHBV and composites during the first heating and first cooling scans are shown in Figure 5. Neat PHBV showed a single melting peak. Its crystallinity is calculated to be 61.4%, which is close to the value measured in an earlier study by using wide-angle X-ray diffraction (58.1%) [42]. While the MB10 composite showed similar crystallinity to the neat PHBV, the crystallinity of the PHBV/MB20 and PHBV/MB30 decreased (Table 2). Furthermore, all three composites showed a broad melting trough with two components: either two peaks or a peak and a shoulder. The temperatures of the peak or the transition of the shoulder at the lower temperature are shown as *T*_m1_ in Table 2, while the melting peak of PHBV and the temperatures of the second peak or transition are represented as *T*_m2_. The *T*_m1_ showed a slight decrease from PHBV/MB10 to PHBV/MB30, whereas the *T*_m2_ of the composites was the same as that of the neat PHBV. The low-temperature component, *T*_m1_, can be attributed to the melting of the unstable imperfect crystals. The more thermally stable perfect crystals melted at the same temperature as the neat PHBV. The imperfect crystals may have also experienced melting-recrystallization during the DSC heating. 

As shown and discussed earlier, biocarbon is porous. The pores can trap air and moisture. Consequently, this can degrade PHBV through thermo-oxidation degradation during processing. Moreover, it is well understood that PHBV is sensitive to moisture [43]. PHBV molecular chains can react with water, resulting in chain scission [9]. The same phenomenon has also been reported in the recent study of PLA filled with biocarbon obtained from spent ground coffee [22]. The appearance of twin endothermic peaks was observed in composites filled with 2.5% to 7.5% biocarbon prepared by melt-processing, but not in the samples prepared by solvent casting. Rheology analysis in that work confirmed the degradation of PLA during melt-processing, as compared to solvent casting.

The addition of large amounts of biocarbon particles can reduce the PHBV’s chain mobility, resulting in lower crystallinity. For example, a previous study of PHB blended with amorphous chitin showed that increasing the chitin contents in the composites led to the gradual flattening of the melting peaks, which disappeared completely at and above 50/50 blending ratio [44]. The study showed that the crystalline structure of PHB was not changed by the blending of chitin. The effect was caused by the intermolecular interactions between PHB and chitin, which restricted the chain movement of PHB molecules. In a different study, the addition of carbon nanotubes (CNTs) had a nucleating effect on PHBV, up to a filler content of 7%. However, at 10% loading, a reverse trend in the crystallinity was observed [42]. In the present work, the effect on polymer chain mobility reduction became more prominent at higher filler contents. Furthermore, as a porous material, biocarbon has a large distribution in its pore sizes, ranging from micro to macro pores [45]. Ikram et al. found, in their study of PP composites with wood and biocarbon, that the biocarbon may absorb molten polymer chains [46]. This can lower the regularity of the polymer’s molecular assembly, further reducing its crystallinity. In addition, the DSC cooling scan in Figure 5b shows that there was a slight decrease of the crystallization temperature, *T*_c_, for the composites. This confirms that the biocarbon particles did not have a nucleating effect on the PHBV.

### 3.4. Thermal Stability

**Figure 6:**
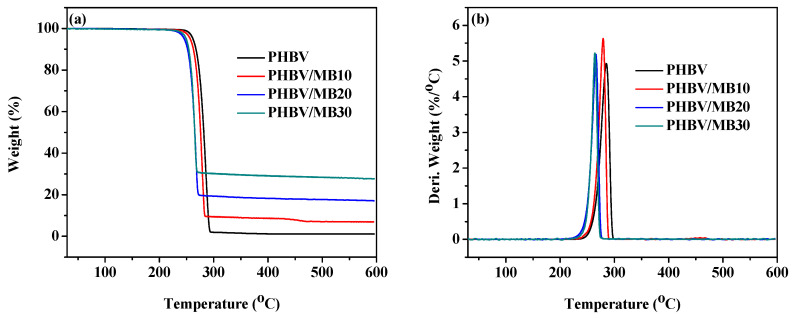
Thermal stability of the PHBV and PHBV/MB composites: (**a**) TGA curves; (**b**) Derivative weight against temperature.

The TGA curves of the PHBV and PHBV/MB composites are shown in Figure 6. The temperatures at 5% weight loss (*T*_5%_) and at the peak of the derivative weight (*T*_max_) are calculated and shown in Table 3. One-step thermal degradation was observed for the neat PHBV and PHBV/MB composites. PHBV is known to degrade thermally by a random one-step β-elimination chain scission reaction [47]. At 10% biocarbon loading, there was a slight decrease of the *T*_5%_ and *T*_max_. The decrease became more prominent with PHBV/MB20 and PHBV/MB30. At 30 wt % filler loading, the *T*_5%_ decreased from 262 °C to 250 °C and *T*_max_ from 285 °C to 264 °C, which is about 20 degrees lower than that of PHBV.

Although biocarbon is a thermally stable material, the phenomenon of high amount of biocarbon reducing the thermal stability of its composites has been reported by many studies, with different polymers such as PP and PLA [19,20,22,48]. Homogeneous dispersion of fillers at low filler contents has been found to improve the thermal stability of polymers. For example, PHBV-grafted multi-walled carbon nanotubes, metal oxides such as ZnO and MgO, silica, and layered silicate have been shown to improve the thermal stability of PHAs slightly [42,49,50,51,52]. However, the increase of thermal stability with the addition of organically modified clay in PHB started to reverse when the particle content was increased from 5 wt % to 10 wt % [53]. In a recent study of talc-reinforced PLA composites, although the composites with 5, 10, and 30 wt % talc showed slightly higher on-set degradation temperatures than PLA, the addition of 40 wt % talc decreased the on-set degradation temperature by 24 degrees [54]. Mechanical and morphological analysis in that study showed the agglomeration of the particles and the presence of voids in and around the particle clusters. These voids, which were also found in the present work, can facilitate the permeation and diffusion of degradation products. In addition, the lower crystallinity of the MB20 and MB30 composites might have also contributed to the slight decrease in thermal stability.

### 3.5. Heat Deflection Temperature (HDT)

**Figure 7:**
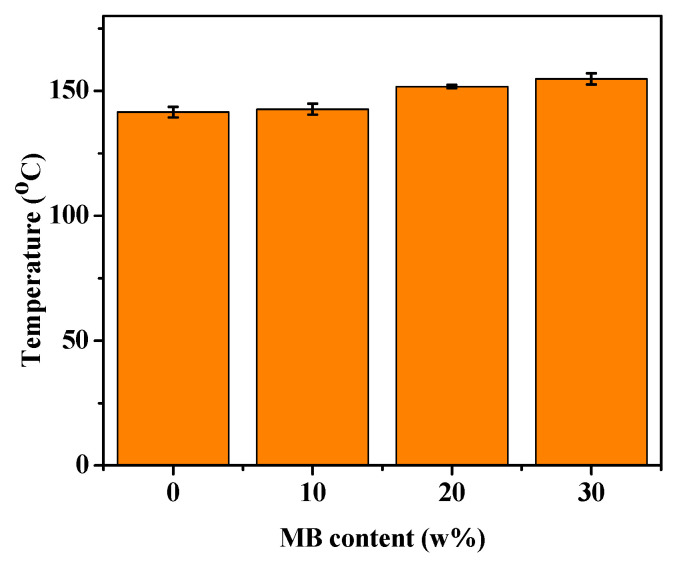
HDT of the PHBV and PHBV/MB composites.

HDT indicates how well a material can withstand structural deformation under a fixed load at elevated temperatures. Higher HDT is often desired in practical applications of polymer composites. Figure 7 shows that the HDT of PHBV was about 142 °C, and increased with the addition of the biocarbon. With 30 wt % biocarbon, the HDT reached 155 °C, about 13 degrees higher than that of PHBV. This increase can again be attributed to the polymer chain movement being restricted by the addition of filler [55]. The improvement of the polymer HDT with the addition of *Miscanthus* biocarbon has also been reported for polyamide composites [27]. As discussed earlier, the pores of the biocarbon might absorb the PHBV chains. This can further hamper the mobility of PHBV molecules, especially in the amorphous phase. Consequently, a higher temperature is required to deform the material, resulting in higher HDT.

### 3.6. Coefficient of Linear Thermal Expansion (CLTE)

**Figure 8:**
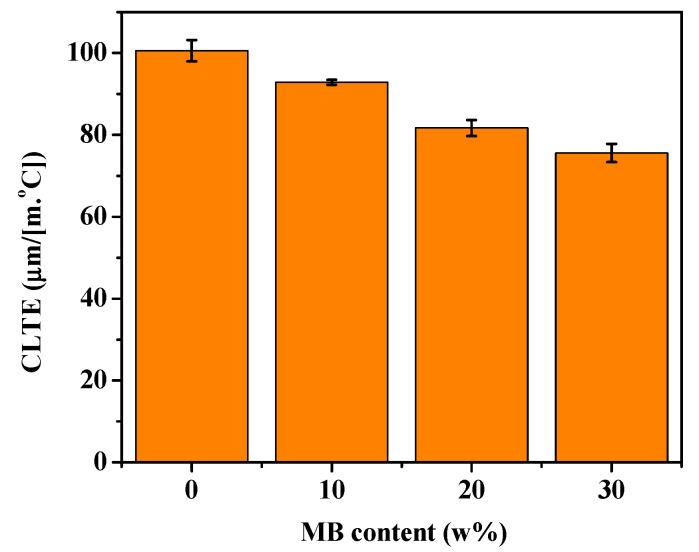
CLTE of the PHBV and PHBV/MB composites.

CLTE is an important parameter in measuring a material’s ability to maintain its dimensional stability at high temperature. The CLTE of a polymer composite depends on the CLTE values of all its components, and their interaction [56]. The CLTE values of the PHBV/MB composites were tested in the flow direction and shown in Figure 8. The neat PHBV showed a CLTE of 100.6 μm/(m·°C). A gradual reduction of the CLTE values was observed for the PHBV/MB composites with increasing MB contents. This corroborates the observation of polymer chain mobility reduction by the biocarbon throughout this study.

## 4. Conclusions

In this research, biobased composites were prepared from biodegredable PHBV and the biocarbon obtained from *Miscanthus*, a perennial grass. The introduction of the biocarbon resulted in an increase in the stiffness, with higher filler contents showing higher modulus, but a decrease in the tensile and flexural strength and elongation-at-break. For example, the composite with 30 wt % biocarbon showed a 46% increase in Young’s modulus, and a 14% decrease in tensile strength. The increase in modulus can be attributed to the stiffness of the fillers, which, however, also acted as stress concentrators, resulting in lower strength. The incorporation of the biocarbon particles reduced the tensile and flexural toughness of the polymer.. Adding high contents of the particles caused increased agglomeration and formation of voids in the composite. The restriction of the polymer chain mobility by the biocarbon is evidenced in the study of thermal behavior, and the testing of HDT and CLTE. DSC analysis showed that the biocarbon did not work as a nucleating agent for PHBV, and higher amounts of biocarbon reduced the crystallinity. The biocarbon caused some degradation and a slight decrease of the thermal stability of PHBV. On the other hand, the HDT was increased and CLTE decreased, indicating enhanced dimensional stability thanks to the addition of the rigid biocarbon particles. These results show the potential of biocarbon derived from biomass as a biobased filler for biodegradable polymers, as well as the need for future work to enhance the adhesion between the biocarbon and polymer in order to improve the composites properties. 

## Figures and Tables

**Table 1:** Tensile properties of the PHBV and its biocarbon composites.

	Young’s Modulus (MPa) (S.D. ^1^)	Tensile Strength (MPa) (S.D.)	Elongation-at-Break (%) (S.D.)
PHBV	3587 (311)	38.3 (1.1)	2.01 (0.37)
PHBV/MB10	3741 (231)	32.2 (1.6)	1.12 (0.08)
PHBV/MB20	4691 (272)	33.1 (2.4)	0.99 (0.19)
PHBV/MB30	5240 (164)	32.8 (0.8)	0.89 (0.07)

^1^ S.D.—standard deviation.

**Table 2:** The melting and crystallization temperatures (*T*_m1_, *T*_m2_ and *T*_c_) and the degree of crystallinity (*χ*_c_) of the PHBV and PHBV/MB composites measured with DSC.

	*T*_m1_ (°C)	*T*_m2_ (°C)	*χ*_c_ (%)	*T*_c_ (°C)
PHBV	n.a.	174	61.4	124
PHBV/MB10	172	175	61.3	121
PHBV/MB20	171	174	50.0	122
PHBV/MB30	170	174	56.6	124

**Table 3:** Characteristic thermal degradation temperatures of the PHBV and PHBV/MB composites.

	*T*_5%_ (°C)	*T*_max_ (°C)
PHBV	262	285
PHBV/MB10	257	279
PHBV/MB20	246	266
PHBV/MB30	250	264

*T*_5%_—temperature at 5% weight loss; *T*_max_—temperature at the maximum rate of weight loss.
